# Prior Drug-Related Criminal Charges and Risk for Intimate Partner Violence Perpetration Among Authorized Purchasers of Handguns in California

**DOI:** 10.1177/08862605221078811

**Published:** 2022-03-25

**Authors:** Rocco Pallin, Mona A. Wright, Elizabeth A. Tomsich, Garen J. Wintemute, Susan Stewart, Rose M. C. Kagawa

**Affiliations:** 1 Violence Prevention Research Program, Department of Emergency Medicine, School of Medicine, 8789 University of California, Davis, Sacramento, CA, USA; 2 Department of Population Health and Reproduction, School of Veterinary Medicine and Department of Public Health Sciences, School of Medicine, 8789 University of California, Davis, Sacramento, CA, USA

**Keywords:** intimate partner violence, firearm purchase, drug use, drug sale, survival analysis, arrest and conviction, firearm prohibitions

## Abstract

Intimate partner violence (IPV) is a considerable public health problem in the US, and evidence suggests that both drugs and firearms contribute to the risk of IPV and its severity. This study uses a retrospective, longitudinal cohort design to explore the association between past arrests, charges incurred in the legal process, and convictions for drug-related crimes, and risk of future arrest for IPV among legal handgun purchasers. The cohort included all legal purchasers of handguns in California in 2001 between the ages of 21 and 49 (*n* = 79,678), 156 of whom had pre-purchase drug charges and post-purchase IPV charges. We used Cox proportional hazards regression with age at time of handgun purchase, sex, and race/ethnicity, and an array of community characteristics as covariates. Over the study period (2001-2013), in comparison to handgun purchasers who had no charges or convictions prior to their index purchase, risk for future IPV arrest was increased for purchasers whose only prior charges were drug-related (aHR = 3.4 [95% CI: 2.4-4.9]) and purchasers who had both prior drug- and non-drug related charges (aHR = 4.9 [95% CI: 4.1-6.0]). The magnitude of the risk ratio was greater when multiple drug types were involved and when the person had been charged with both the use and sale of drugs. Our findings suggest that, among legal handgun purchasers, prior drug charges are associated with future risk of IPV arrests or convictions. Given the established link between firearm access and IPV severity and fatality, these findings may inform the development and enforcement of policies that reduce firearm access for those at elevated risk of perpetrating intimate partner violence.

## Introduction

More than one-third of women and nearly 30% of men in the United States experience intimate partner violence (IPV) in their lifetimes, and nearly one in four women and one in seven men experience severe physical violence by an intimate partner (Smith et al., 2017).

Access to firearms intensifies IPV. When an abuser has access to a gun, the risk of homicide increases five times (Campbell et al., 2003). Among the strongest risk factors for intimate partner homicide are firearm access and the perpetrator having made prior threats with a weapon (Spencer & Stith, 2020). Firearms are involved in approximately half of reported IPV homicides in the US (Gollub & Gardner, 2019), and one million US women have been shot or shot at by an intimate partner (Smith et al., 2017; Zeoli et al., 2016).

Firearms are even more commonly used to threaten, coerce, and scare intimate partner victims (Sorenson & Schut, 2016, p. 201; Zeoli et al., 2016). One systematic review suggests that 4.5 million American women alive today have been threatened by an intimate partner with a gun (Sorenson & Schut, 2016). Such nonfatal firearm use can facilitate chronic and intensifying abuse, with longstanding physical and psychological consequences (Sorenson & Schut, 2016).

Existing federal policy seeks to lessen the possibility of known abusers legally gaining access to firearms: those subject to final domestic violence restraining orders and those convicted of misdemeanor domestic violence crimes and felonies are prohibited from purchasing or possessing firearms (Gun Control Act of 1968, 18 U.S.C. §§921-931). State policies governing firearm access by domestic abusers vary widely, but studies suggest that in states where firearm prohibitions accompany domestic violence restraining orders, IPV-related misdemeanors, or convictions for stalking, the odds of IPV-related injury were lower (Willie et al., 2021), and in states where firearm prohibitions accompany domestic violence restraining orders, IPV homicide is reduced (Zeoli & Webster, 2010). Federal policy also includes a prohibition on firearm purchase and possession by “unlawful users of or those addicted to a controlled substance” (Gun Control Act of 1968, 18 U.S.C. §§921-931).

Research has examined the link between drug involvement and violence generally, as well as between substance use, including alcohol, and IPV perpetration and victimization. Growing evidence supports drug use as a risk factor for IPV perpetration (Abrahams et al., 2006; Cafferky et al., 2018; Chermack et al., 2008; Coker et al., 2000; Fals-Stewart et al., 2003; Fergusson et al., 2008; Kyriacou et al., 1999; B. C. Moore et al., 2011; T. Moore et al., 2008; Pryor et al., 2020; Stuart et al., 2008). One study found an estimated odds ratio of 1.7 for intimate partner violence perpetration among those with a substance use disorder relative to those without such a disorder (Zhong et al., 2020). Whether the associations differ depending on drug type remains unclear (Cafferky et al., 2018; Thiessen et al., 2018; Walsh et al., 2016).

There are strong associations between firearm access and IPV severity (Campbell et al., 2003; Sorenson & Schut, 2016; Zeoli et al., 2016), IPV and other forms of firearm violence (Geller et al., 2021; Kivisto & Porter, 2020; Smith et al., 2014; Smucker et al., 2018), and firearm violence and substance misuse (Branas et al., 2016; Hohl et al., 2017; Kuhns et al., 2014; Wintemute, 2015). This study seeks to contribute to the literature on the association between drug involvement and IPV by assessing risk among a cohort of legal handgun purchasers (i.e., individuals who were not subject to prohibitions on ownership at their times of firearm purchase in 2001). We use a longitudinal study design to determine whether and to what extent handgun purchasers’ history of drug-related arrests, charges incurred in the legal process, and convictions for drug offenses (henceforth, “drug charges”) is associated with risk for future IPV perpetration. We also examine the risk of future IPV perpetration by type of drug, whether the arrest or conviction was for drug sale or use, a subject’s number of drug charges, and the time since the most recent drug charge prior to the index (2001) handgun purchase.

## Methods

### Study Population

This study included all legal handgun purchasers in California in 2001 between the ages of 21 (the legal age for handgun purchase in the state) and 49 (*n* = 79,678). We chose the upper bound to reflect the evidence that rates of criminal behavior drop over time and are relatively low after the age of 50 (Loeber & Farrington, 2014). The subjects were identified using the California Department of Justice Dealer’s Record of Sale (DROS) database, which records information on all legal handgun purchases and transfers in the state.

We followed the study population from 10 days after their first 2001 handgun purchase (henceforth the “index purchase”), the first day on which purchased firearms could be acquired due to California’s ten-day waiting period, through the end of 2013. Subjects were considered at risk for IPV perpetration only for as long as we could verify their residence in California, independent of outcome events using the California Death Statistical Master File, California voter registration records, DROS records, and Lexis-Nexis Public Records.

The cohort includes only legal purchasers of handguns and therefore excludes persons with prohibitions on firearm purchase and possession according to federal and/or California law (e.g., persons convicted of felonies prior to their index purchase, persons convicted of domestic violence misdemeanors within 10 years of their 2001 purchase dates, and persons who were subject to active restraining orders). As a result, people with drug offenses in this study sample are not representative of the general population of those with drug offenses. Rather, they are likely representative of people with drug offenses who legally purchased handguns around the turn of the century.

### Exposures and Outcomes

Our primary exposure was arrests, charges incurred in the legal process, and/or convictions for drug offenses (“drug charges”) prior to the index purchase. We categorized subjects into four groups for this analysis, based on charges accrued prior to the index purchase: those with 1) no charges, 2) only non-drug charge(s) (e.g. weapon, alcohol, vandalism, vehicle, forgery, assault, burglary, or robbery charges), 3) only drug charge(s), and 4) both drug and non-drug charges. Secondary exposures included drug type (marijuana vs. other drugs); whether the pre-purchase charges were drug use charges, drug sale charges, or both; the number of pre-purchase drug charges (one vs. more than one); and recency of most recent drug charge (within 2 years of index purchase vs. more than 2 years prior). Examples of secondary exposure charges appear in Appendix A.

Our outcome was being charged with an IPV offense beginning 10 days after firearm purchase, including charges such as battery, rape by force/threat/fear, inflicting corporal injury of a current or former intimate partner, and violation of protective order resulting in physical harm. We included only charges that specifically indicated, per the California penal code, that these crimes were against intimate partners (current or former). We used arrest charges, charges accrued through court processes, and convictions (rather than convictions alone) to more comprehensively capture events of IPV (Maltz, 1984). Evidence suggests that prosecution of IPV is low relative to other types of offenses and that final dispositions of arrests are not always reported to state criminal history databases (Ackerman & Love, 2014; Blumstein, 1982; Holliday et al., 2020; Maltz, 1984). Examples of outcome charges appear in Appendix B.

Criminal history data were obtained using deterministic and probabilistic linking between California’s Criminal History Information System records and DROS records. Both databases contain full name, date of birth, sex, city, zip code, and driver’s license number. When deterministic linkage was not possible, we used LinkPlus version 2.0 to probabilistically link records. The linkage process is described in further detail in (Kagawa et al., 2020).

### Covariates

We included individual- and community-level variables with *p* < 0.25 in the primary and secondary models when all variables were included simultaneously. Individual-level covariates that met this threshold included purchaser’s age at time of purchase, sex, and race/ethnicity. Individual demographics were recorded on the DROS form at the time of purchase. Community-level covariates included the percentage of the census tract population that was white, the percentage of the census tract population that was Black, and the county violent crime rate per 100,000 population in 2001. Community demographic information is sourced from the American Community Survey using annual interpolations for 2001 and the county violence crime rate is based on Uniform Crime Reporting statistics for California. Criminal history information is from the CA DOJ Criminal History Information System.

### Statistical Analyses

We used Cox proportional hazards regression analysis to estimate adjusted hazard ratios (aHRs) with 95% confidence intervals. In the primary model, the exposure was type of charge history (i.e., none, only non-drug, only drug, and both drug and non-drug). Each secondary exposure (e.g. drug type, sale vs. use) was run in a separate model and those who had no criminal history or only non-drug charges were included in the referent group. In secondary models, we also controlled for the presence or absence of a history of non-drug criminal charges. For regressions with multi-level independent variables, we used pairwise comparisons of marginal linear predictions to test the significance of between-group differences in the adjusted hazard ratios (e.g., to compare the variation in aHRs among those whose only drug charges were marijuana charges with those whose drug charges were marijuana and other drug charges). We graphed Schoenfeld residuals and log-log plots to test the proportional hazards assumption. The residuals for the exposure variables, which appeared flat over time, suggest this assumption was met. The residuals for the covariate age appeared to violate this assumption. However, results do not differ meaningfully when including age as a time dependent covariate using interactions (results not shown). Finally, we graphed the Kaplan-Meier curves for all exposures.

We conducted a sensitivity analysis in which we excluded the 40 cohort members whose only drug charges were for marijuana offenses that were illegal in 2001 but policy changes have since removed these offenses from criminal statute.

Statistical significance was assessed at the *p* ≤ 0.05 level (2-sided). Analyses were conducted using Stata, version 15.1 (StataCorp LP, College Station, TX). The Institutional Review Board at the University of California, Davis, approved the study protocol (Wintemute et al., 2016).

## Results

### Study Cohort

Purchasers were overwhelmingly male (91.0%), and two-thirds were white (68.9%). For more than half the cohort (53.0%), the index purchase was their first handgun purchase as per DROS. Demographic characteristics of the cohort are described in Table 1.

**Table 1.** Demographic Characteristics of 2001 Handgun Purchasers.

	Count	Percent
Total	79,678	—
Age		
21-29	25,818	32.4
30-39	27,941	35.1
40-49	25,919	32.5
Gender		
Male	72,522	91.0
Female	7156	9.0
Race/Ethnicity^a^		
White	54,868	68.9
Hispanic	12,190	15.3
Asian	6405	8.0
Black	4298	5.4
American Indian	479	0.6
Other	1409	1.8
Prior handgun purchases		
None	42,241	53.0
One	12,025	15.1
Two or more	25,412	31.9

^a^29 cohort members are missing information on race/ethnicity.

### Cohort’s Pre-Purchase Drug Charge Histories

Three percent of the cohort (*n* = 2554) had one or more drug charges prior to the index handgun purchase. A larger share (13.5%, *n* = 10,738) had pre-purchase histories of non-drug charges only while a much smaller share had drug charges only (0.9%, *n* = 692). A description of pre-purchase drug charges appears in Table 2.

**Table 2.** Cohort Members’ Charge Histories and Types of Drug Charges Prior to 2001 Handgun Purchase. (*N* = 79,678).

	Count	Percent
Charge history		
No charges	66,386	83.3
Non-drug charges only	10,738	13.5
Drug charges only	692	0.9
Drug & non-drug charges	1862	2.3
Type of charges		
Drug charges only		
Marijuana only	102	14.7
Other drug only	539	77.9
Marijuana & other drug	51	7.4
Drug & non-drug charges		
Marijuana only & non-drug charges	251	13.5
Other drug only & non-drug charges	1429	76.7
Marijuana, other drug & non-drug charges	182	9.8

The majority of those with drug charges (86.2%, *n* = 2201) had charges only for drugs other than marijuana, a majority (83.4%) had use-related charges, and three-fifths had one drug charge (vs. more than one) (Table 3). For a large majority of those with drug charges (90.3%), the most recent charge occurred more than 2 years prior to the index handgun purchase.

**Table 3.** Characteristics of Cohort Members’ Pre-Purchase Drug Charges. (*N* = 79,678).

	Count	Percent
Total with drug charges	2554	—
Pre-purchase history of drug use and sale charges^a^		
Use only	1703	67.3
Sale only	399	15.8
Use & sale	428	16.9
Number of pre-purchase drug charges		
One	1551	60.7
More than one	1003	39.3
Time between 2001 purchase and most recent drug charge		
2 years or fewer	248	9.7
More than 2 years	2306	90.3

^a^24 purchasers had drug charges that could not be classified as use or sale and are excluded.

Two percent of the cohort (*n* = 1601) had a charge for an IPV offense in the follow-up period. Nearly 10% (9.7%, *n* = 156) of those with an IPV charge in the follow-up period had drug charges prior to their 2001 handgun purchase. Six percent (*n* = 156) of cohort members with pre-purchase drug charges had a subsequent IPV charge.

### Proportional Hazards Regression Analyses

**Figure 1.**
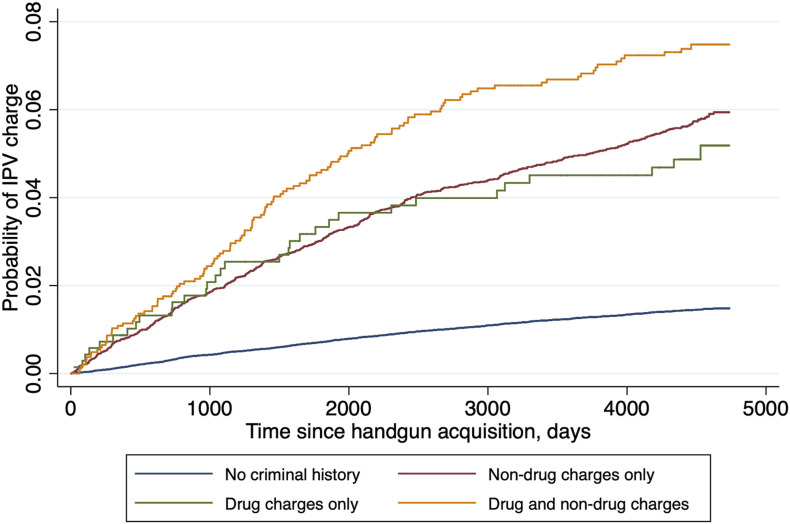
Time to IPV charge after handgun acquisition by pre-purchase drug-related criminal history. (*N* = 79,046).

We found a history of both drug and other charges was associated with the highest risk of future IPV charge (aHR = 4.9, 95% CI = 4.1-6.0) relative to those with no criminal history, after controlling for the individual- and community-level covariates described previously (Table 4, Figure 1). The hazards of IPV charges among those with pre-purchase drug charges only and non-drug charges only were smaller, though remained elevated relative to those with no criminal history, and the Kaplan Meier curve suggests a similar risk trajectory for these two groups.

**Table 4.** Hazard of Post-Purchase IPV Charge by History of Drug Charges. (*N* = 79,046).

	aHR	95% CI	*p* value
Drug charges only	3.4	2.4–4.9	<0.001
Non-drug charges only	3.4	3.1–3.8	<0.001
Drug & non-drug charges	4.9	4.1–6.0	<0.001
No charges	Ref	—	

Notes: the model controlled for purchaser’s age at time of purchase, sex, and race/ethnicity, percentage of the census tract population that was white, percentage of the census tract population that was Black, and county violent crime rate per 100,000 population in 2001. The number of cohort members in this model is less than the total 79,687 because some members were missing the community-level covariates.

**Figure 2.**
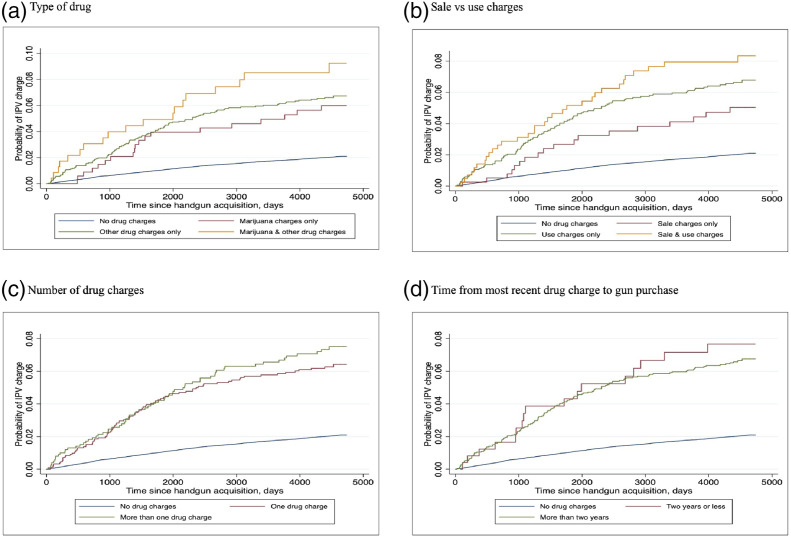
Time to IPV charge after handgun acquisition by pre-purchase drug-related criminal history and characteristics of drug charges. (*N* = 79,046).

In our secondary analyses, we found the hazard of future IPV charges associated with pre-purchase drug charges varied by the characteristics of those charges (Table 5, Figure 2). Subgroups at greatest risk, relative to those with no criminal history or with only non-drug charges, included those with other drug offenses (alone or in combination with marijuana charges), use charges (alone or in combination with sale charges), and those whose most recent drug charge was more than two years from the index purchase. The risk of subsequent IPV perpetration for those with only marijuana charges, only sale charges, and those whose most recent drug charge was within two years of the index purchase was not statistically distinguishable from the referent group. Finally, there was no apparent dose response relationship between the number of drug charges and IPV risk.

**Table 5.** Hazard of IPV Charge Associated With History of Drug Charges Compared to Those With No Drug Charge History, By Drug Charge Characteristics. (*N* = 79,046).

	aHR	95% CI	*p* value
Type of drug			
Marijuana only	1.3	0.8–2.1	0.234
Other drug only	1.7	1.4–2.1	<0.001
Marijuana & other drug	2.2	1.4–3.5	0.001
Use charge or sale charge			
Use only	1.7	1.4–2.1	<0.001
Sale only	1.1	0.7–1.8	0.681
Use & sale	2.0	1.4–2.8	<0.001
Number of charges			
One charge	1.6	1.3–2.0	<0.001
More than one charge	1.8	1.4–2.3	<0.001
Time between purchase and most recent drug charge			
Two years or less	1.4	0.9–2.3	0.167
More than two years	1.7	1.4–2.1	<0.001

Notes: Cohort members with no criminal history or with only non-drug charges were included in the referent group. The differences in the aHRs among groups within categories above (e.g., type of drug, use charge or sale charge, etc.) were not statistically significant at *p* < 0.05. All models controlled for purchaser’s age at time of purchase, sex, race/ethnicity, presence of pre-purchase non-drug charges, percentage of the census tract population that was white, percentage of the census tract population that was Black, and county violent crime rate per 100,000 population in 2001. A different model was run for each exposure group. The number of cohort members in this model is less than the total 79,687 because some members were missing the community-level covariates.

### Sensitivity Analysis

The cohort included 40 people whose only drug charges were for marijuana offenses that were illegal in 2001 but, through policy changes, have since been removed from criminal statute. We conducted a separate sensitivity analysis that excluded this group from the model, which resulted in very similar adjusted hazard ratios as the main model.

## Discussion

We found, for legal purchasers of handguns, that the risk of a future IPV charge was significantly elevated for those with only pre-purchase drug arrest(s) or conviction(s) compared to those with no criminal history. Further, cohort members with both drug and non-drug charges prior to purchase were at highest risk—nearly five times the risk compared to those with no criminal history prior to purchase—for subsequent IPV charges. These results suggest that drug charges alone as well as non-drug charges alone increase risk for IPV arrests or convictions, and in combination, the risk for future perpetration of IPV is further increased.

In secondary models, we found that different types of drug charges may variably affect the magnitude of the increased risk for future IPV compared to the cohort members without drug charges. We observed that a history that includes charges for other drugs (i.e., drugs aside from marijuana) may be associated with higher risk than a history that includes only charges for marijuana. In prior studies, however, marijuana has been associated with IPV perpetration (Cafferky et al., 2018). Because we could not identify the specific type of drugs included in the “other drug” category, our results cannot provide additional evidence on use of specific illicit drugs as a risk factor for IPV perpetration.

The fact that the group with both marijuana and other charges is at highest risk may reflect a dose-response phenomenon, that is, that increased breadth of prior criminal activity may be associated with an increase in future risk. In fact, the mean number of charges (of any kind) for those with both marijuana and other drug charges was 7.4. For comparison, those whose drug charges were only for marijuana offenses had a mean 3.2 charges and those whose drug charges were only for other drug offenses had a mean of 4.6 charges. Having both marijuana and other drug charges may signal more involvement in criminal activity and thus higher risk relative to having one or the other (in a similar way as a history of both drug and non-drug charges is associated with higher risk, as the main model results suggest). Preceding studies with this same cohort similarly found that those with a variety of charges had greater risk for future criminal charges compared to those that had only one type of criminal charge (e.g., alcohol- or IPV-related charges) before purchase (Kagawa et al., 2020; Tomsich et al., 2021).

We also observed that a history that includes use-related drug charges (i.e., use charges only or use and sale charges) may be at higher risk for IPV than one with only sale-related drug charges. Further research would be useful for discerning differences in risk by drug use and sale, as the existing literature suggests a more consistent association between drug sales and firearm violence (though not specifically IPV) than drug use and firearm violence (McGinty et al., 2016).

Our findings fit within the existing literature, including that which finds involvement with drugs associated with perpetrating IPV (Abrahams et al., 2006; Cafferky et al., 2018; Chermack et al., 2008; Coker et al., 2000; Fals-Stewart et al., 2003; Fergusson et al., 2008; Kyriacou et al., 1999; B. C. Moore et al., 2011; T. Moore et al., 2008; Pryor et al., 2020; Stuart et al., 2008; Zhong et al., 2020) and that which suggests that arrests for nonviolent crimes serve as a risk marker for IPV perpetration (Fox et al., 2020). Further work could inform policy by examining the relationship between drug conviction alone and future perpetration of IPV, as arrests do not indicate guilt and are not often used as the basis for policy.

Note also that drug arrests and convictions are subject to racial bias. Black and Latinx people, especially males, experience disproportionately high rates of drug-related arrest, conviction, and prison sentencing (Camplain et al., 2020; L. D. Moore & Elkavich, 2008; Reuter, 2013). Because of this inequity, the extent to which arrest for drug charges serves as a risk marker for IPV likely varies for people of different race-ethnicities. That is, if Black persons are more likely to be charged with a drug offense than are white persons who engage in the same drug-related behavior, we will incorrectly assign disproportionately high risk (and firearm prohibition) to Black persons. For this reason, firearm policy based on drug charges and convictions is likely to have inequitable impacts on racial/ethnic groups.

This study’s cohort is unique in that no study subject could have been convicted of a felony, convicted of a misdemeanor domestic violence crime in the 10 years prior to the index purchase, a respondent to an active domestic violence protective order, deemed an “unlawful user of” or “addicted to” a controlled substance according to federal law (with rare exceptions for deferral agreements), nor subject to any other federal firearm prohibition. These selection criteria likely bias our results towards the null. In other states, where fewer prohibitions on firearm purchase and possession may mean more people with higher likelihood of perpetrating violence can purchase and possess firearms, the association between drug charges and IPV perpetration among purchasers of firearms may be more pronounced.

These results may inform policy and enforcement, especially considering that unlawful users of controlled substances are already prohibited from purchasing and possessing firearms at the federal level. There may be opportunities to reduce the incidence and severity of intimate partner violence through the creation and improved enforcement of firearm purchase and possession restrictions on those with criminal histories that include drug or non-drug charges, and particularly for those with more than one type of pre-purchase charge (i.e., those with drug and non-drug charges). Both state governments and federal policies aim to disallow gun purchase and possession among persons at elevated risk, including those at risk for perpetrating IPV. Efforts to enforce such prohibitions are underway in some states, including programs to enforce the removal of firearms from those who become prohibited from owning firearms after purchasing them (Pear et al., 2021) or removal of firearms at the time domestic violence restraining orders are served (Zeoli et al., 2019).

Nonetheless, IPV is a complex issue, and reducing the incidence and severity requires attention to the range of risk factors for and systemic drivers of IPV perpetration. While maximally reducing access to firearms by those at risk of IPV perpetration deserves attention, enforcing firearm prohibitions alone will be insufficient to achieve complete prevention. Research suggests that abusers’ access to firearms markedly increases the risk that IPV events result in fatalities and that abusers with guns tend to inflict more severe abuse relative to those without (Campbell et al., 2003). However, our results suggest those with drug-related charges represent a small proportion of those who were charged with IPV after purchase. There is also the possibility that being without firearms will not deter abusers, especially chronic abusers, as well as the possibility of means substitution (i.e., an abuser’s use of knives or other readily accessible weapons should firearms become unavailable to the abuser). In fact, gun owners may be as likely to threaten their partners with knives as non-gun owners (Rothman et al., 2005). A range of primary and secondary prevention strategies aimed at reducing IPV by affecting individual behaviors as well as community and societal factors that contribute to risk, such as developing protective environments, enhancing family economic supports, and supporting survivors, along with other methods to limit access to firearms by perpetrators of IPV, deserve attention.

### Limitations

This study should be interpreted in light of several limitations. First, IPV is underreported; evidence suggests that police are not notified in nearly half of nonfatal domestic violence incidents (Ackerman & Love, 2014; Holliday et al., 2020). Thus, our criminal records data, which includes arrests and convictions, certainly undercounted IPV perpetration. Second, drug charges do not perfectly capture pre-purchase drug-related activity and drug use. Third, prohibited persons, who may be at higher risk for committing violence, were excluded from this sample of legal purchasers. Federal and state firearm purchase and possession prohibitions related to domestic violence, which prohibit those convicted of felonies, misdemeanor domestic violence crimes, and those who are respondents to domestic violence protective orders, may mean a group at particularly high risk for IPV perpetration was excluded. Additionally, federal law prohibits firearm ownership by “unlawful users of or those addicted to a controlled substance.” The implications of the “unlawful user” prohibition for our study are unknown; drug convictions or court actions often result in drug diversion programs, which can clear a conviction from a person’s record. However, nationwide, history of drug charges is among the top reasons for firearm purchase denial (Brooks, 2021). Because our cohort included only legal purchasers of handguns in 2001, those with IPV convictions or drug convictions that designate them “unlawful users” of controlled substances were excluded from the sample, limiting us from generalizing the association between drug-related charges and IPV perpetration to a wider population. Our cohort did not include purchasers above age 50, which likely also limits the generalizability of these results. Evidence suggests that risk for perpetration of IPV peaks in the mid-20s and declines in the mid-30s (Gelles & Straus, 1988), suggesting the associations observed here would be smaller in magnitude or non-existent among older more infrequent offenders. Finally, our study included legal handgun purchasers in one state, and cohort members were overwhelmingly male and white. The generalizability of these results to other populations is limited.

Our sample sizes for the secondary analyses (examining drug use vs. sale, types of drugs, etc.) were also limiting as they are small relative to the entire cohort. Nevertheless, we were able to estimate hazard ratios with adequate precision.

Our analyses were also limited with respect to our outcome of interest. We were unable to separate IPV charges that involved firearms from those that did not. Such distinction would allow for a deeper examination of the relationship between firearms and IPV among legal gun purchasers with risk factors such as prior drug charges. Further, we may not have captured all intimate partner outcome events as we only included charges that specifically indicated, per the California Penal Code, the crime (e.g., battery, rape by force/threat/fear, inflicting corporal injury) was against an intimate partner. Because of the relatively low incidence of post-purchase IPV perpetration in our cohort, we were not able to stratify results by characteristics of IPV charges. Similarly, because of the relatively low frequency of charges, we were unable to conduct analyses using combinations of characteristics of drug charges (e.g., use of marijuana vs. sale of marijuana). We may have introduced measurement error as we manually classified drug use and sale charges.

### Implications and Conclusions

There is an established link between drug-related criminal history and IPV perpetration. Evidence suggests that firearms relate to IPV as they are often used to threaten and coerce victims of IPV, and that firearm access increases the likelihood of fatal IPV. This study identifies arrests, charges incurred in the legal process, and/or convictions for drug offenses as a risk factor for IPV perpetration among legal handgun purchasers. These results suggest that additional enforcement of existing drug-related prohibitions could reduce the risk of subsequent IPV or that the prohibition’s definition itself may be problematic. Policymakers may benefit from a more nuanced examination of the association between drug charges and future IPV perpetration by examining different cohorts and by using more detailed characterizations of drug charges to identify associations between use and sale of specific types of drugs and risk for IPV perpetration.

## Appendix Material for Prior drug-related criminal charges and risk for intimate partner violence perpetration among authorized purchasers of handguns in California

### Appendix A

**Table:** Example exposure charges: Top 20 drug-related offenses (92.0% of all drug-related offenses) by drug type and usage.

California Offense			Drug Type		Drug Usage	
Code^a^	Section	Description	Marijuana	Other drug	Use	Sale
H	11550(a)	Use/under influence of controlled substance		X	X	
H	11377(a)	Possess controlled substance		X	X	
H	11350(a)	Possess controlled substance		X	X	
H	11364	Possess control substance paraphernalia		X	X	
H	11359	Possess marijuana for sale	X			X
H	11358	Plant/cultivate/etc. marijuana/hash	X			X
H	11360(a)	Give/etc. marijuana over 1 oz/28.5 grm	X			X
H	11378	Possess control substance for sale		X		X
H	11357(a)	Possess concentrated cannabis	X		X	
P	647(f)	Disorderly conduct: Under influence of toluene		X	X	
H	11550(b)	Use/under influence of specified control substance		X	X	
H	11351(a)	Possess narcotic control substance for sale		X		X
H	11352(a)	Transport/sell control substance		X		X
B	4149	Possess hypodermic needle/syringe		X		X
H	11379(a)	Transport controlled substance		X		X
H	11351.5	Possess/purchase cocaine base for sale		X		X
H	11,910	Possess dangerous drugs		X	X	
V	23105(a)	DUI: Any drug		X	X	
V	23222(b)	Possess marijuana 1 oz or less while driving	X		X	
B	4143(a)	Possess hypodermic needle/syringe		X	X	

^a^California code: H = Health and Safety, P = Penal, B = Business and Professional, V = Vehicle.

## Appendix B

**Table:** Intimate partner outcome offenses

Code^a^	Section	Description
P	166(c)(2)	Violation of protective order resulting in physical injury
P	166(c)(4)	Violation of protective order with prior
P	243(e)(1)	Battery spouse/ex spouse/date/etc
P	242-243(e)	Assault and battery: Spouse/ex spouse/date/etc.
P	262(a)(1)	Rape of spouse by force/threat/fear
P	273.5(a)	Inflict corporal injury: Spouse/cohabiting sexual partner/date/etc.
P	273.5(e)(1)	Inflict corporal injury resulting in traumatic condition
P	273.6(a)	Violation of protective order to prevent domestic violence

^a^California code: P = Penal.
